# Management of the critically poisoned patient

**DOI:** 10.1186/1757-7241-17-29

**Published:** 2009-06-29

**Authors:** Jennifer S Boyle, Laura K Bechtel, Christopher P Holstege

**Affiliations:** 1 Division of Medical Toxicology, Department of Emergency Medicine, University of Virginia School of Medicine, Charlottesville, Virginia, USA; 2 Division of Medical Toxicology, Department of Pediatrics, University of Virginia School of Medicine, Charlottesville, Virginia, USA

## Abstract

**Background:**

Clinicians are often challenged to manage critically ill poison patients. The clinical effects encountered in poisoned patients are dependent on numerous variables, such as the dose, the length of exposure time, and the pre-existing health of the patient. The goal of this article is to introduce the basic concepts for evaluation of poisoned patients and review the appropriate management of such patients based on the currently available literature.

**Methods:**

An unsystematic review of the medical literature was performed and articles pertaining to human poisoning were obtained. The literature selected was based on the preference and clinical expertise of authors.

**Discussion:**

If a poisoning is recognized early and appropriate testing and supportive care is initiated rapidly, the majority of patient outcomes will be good. Judicious use of antidotes should be practiced and clinicians should clearly understand the indications and contraindications of antidotes prior to administration.

## Introduction

Poisoning emergencies commonly present to emergency departments. The clinical effects encountered in poisoned patients are dependent on numerous variables, such as the dose, the length of exposure time, and the pre-existing health of the patient. If a poisoning is recognized early and appropriate supportive care is initiated rapidly, the majority of patient outcomes will be good. The goal of this article is to introduce the basic concepts for evaluation and appropriate management of the poisoned patient.

### Resuscitation/Initial management

The initial approach for evaluating the critically poisoned patient centers on thorough assessment, appropriate stabilization and supportive care [1]. It is important to consider a broad differential diagnosis that includes both toxicological and non-toxicological emergencies to avoid prematurely excluding potentially serious conditions. For example, an obtunded patient who smells of alcohol could also be harboring an intracranial hemorrhage and an agitated patient believed to be anticholinergic may in fact be encephalopathic due to a metabolic or infectious illness.

Aggressive resuscitation is often required for the patient presenting with a toxicologic emergency. This follows a standard "ABC" approach with attention to "airway, breathing and circulation" respectively. The critically poisoned patient may present with central nervous system (CNS) depression or coma necessitating intubation in order to adequately protect the airway and reduce aspiration risk. Ventilatory drive may also be impaired resulting in CO_2 _narcosis with subsequent acidosis and mental status deterioration which may further increase risk for aspiration. Often this deterioration can be unrecognized in the patient placed on high flow oxygen because O_2 _saturation measures may remain adequate despite significant ventilatory failure. In assessing and managing circulatory status, appropriate intravenous access is essential. All severely poisoned patients should have at least one large bore peripheral intravenous catheter, and hypotensive patients should have a second intravenous line placed in either the peripheral or central circulation. Should vasopressor support be required, attention should be given to the specific poison as the mechanism producing hypotension may help direct the vasopressor selection. Agents with peripheral alpha antagonism, such as the atypical antipsychotic olanzapine, may respond well to direct alpha stimulation with phenylephrine [1]. Severe hypotension from tricyclic antidepressants, believed to be in part caused by depletion of biogenic amines, may respond to repletion with a direct alpha agonist such as norepinephrine when other agents such as the mixed alpha agonist dopamine have been ineffective [2].

### Diagnostic approach

#### Toxidromes

Identification of the constellation of signs and symptoms that define a specific toxicologic syndrome, or "toxidrome", may narrow a differential diagnosis to a specific class of poisons [3]. Descriptions of selected toxidromes may be found in Table 1. Many toxidromes have several overlapping features. For example, anticholinergic findings are highly similar to sympathomimetic findings, with one exception being the effects on sweat glands: anticholinergic agents produce warm, flushed dry skin, while sympathomimetic produce diaphoresis. Toxidrome findings may also be affected by individual variability, co-morbid conditions, and co-ingestants. For example, tachycardia associated with sympathomimetic or anticholinergic toxidromes may be absent in a patient who is concurrently taking beta antagonist medications. Additionally, while toxidromes may be applied to classes of drugs, some individual agents within these classes may have one or more toxidrome findings absent. For instance, meperidine is an opiate analgesic, but does not induce miosis that helps define the "classic" opiate toxidrome. When accurately identified, the toxidrome may provide invaluable information for diagnosis and subsequent treatment, although the many limitations impeding acute toxidrome diagnosis must be carefully considered.

**Table 1:** Toxidromes

**Toxidrome**	**Site of Action**	**Signs and symptoms**
Opioid	opioid receptor	sedation, miosis, decreased bowel sounds, decreased respirations

Anticholinergic	muscurinic acetylcholine receptors	altered mental status, sedation, hallucinations, mydriasis, dry skin, dry mucous membranes, decreased bowel sounds and urinary retention

Sedative-hypnotic	gamma-aminobutyric acid receptors	sedation, normal pupils, decreased respirations

Sympathomimetic	alpha and beta adrenergic receptors	agitation, mydriasis, tachycardia, hypertension, hyperthermia, diaphoresis

Cholinergic	nicotinic and muscurinic acetylcholine receptors	altered mental status, seizures, miosis, lacrimation, diaphoresis, bronchospasm, bronchorrhea, vomiting, diarrhea, bradycardia

Serotonin syndrome	serotonin receptors	altered mental status, tachycardia, hypertension, hyperreflexia, clonus, hyperthermia

#### Hyperthermic syndromes

Toxin induced hyperthermia syndromes include sympathomimetic fever, uncoupling syndrome, serotonin syndrome, neuroleptic malignant syndrome, malignant hyperthermia, and anticholinergic poisonings [4]. Sympathomimetics, such as amphetamines and cocaine, may produce hyperthermia due excess serotonin and dopamine resulting in thermal deregulation [5]. Treatment is primarily supportive and may include active cooling and administration of benzodiazepine agents. Uncoupling syndrome occurs when the process of oxidative phosphorylation is disrupted leading to heat generation and a reduced ability to aerobically generate Adenosine-5'-triphosphate (ATP). Severe salicylate poisoning is a characteristic toxin that has been associated with uncoupling [6]. The development of hyperthermia in the salicylate poisoned patient is an indicator of advanced poisoning that will likely require dialysis. Serotonin syndrome occurs when there is a relative excess of serotonin at both peripheral and central serotonergic receptors [7]. Patients may present with hyperthermia, alterations in mental status and neuromuscular abnormalities (rigidity, hyperreflexia, clonus) although there may be individual variability in these findings. It is associated with drug interactions such as the combination of monoamine oxidase inhibitors and meperidine, but may also occur with single agent therapeutic dosing or overdose of serotonergic agents. The serotonin antagonist cyproheptadine has been advocated to treat serotonin syndrome in conjunction with benzodiazepines and other supportive treatments such as active cooling. However, cyproheptadine may only be administered orally and its true efficacy is not well known which limits its overall utility. Neuroleptic malignant syndrome is a condition caused by relative deficiency of dopamine within the central nervous system [8]. It has been associated with dopamine receptor antagonists and the withdrawal of dopamine agonists such as levodopa/carbidopa products. Clinically it may be difficult to distinguish from serotonin syndrome and other hyperthermic emergencies. Bromocriptine, amantadine, and dantrolene have been utilized in some reports, but true efficacy has not been fully delineated. Malignant hyperthermia occurs when genetically susceptible individuals are exposed to depolarizing neuromuscular blocking agents or volatile general anesthetics [9]. Treatment consists of removing the inciting agent, supportive care, and dantrolene administration. Finally, anticholinergic poisoning may result in hyperthermia through impairment of normal cooling mechanisms such as sweating. Supportive care including active cooling and benzodiazepines are the primary treatments for this condition. Overall, differentiating between the toxic hyperthermic syndromes may be challenging and additional causes of hyperthermia such as heat stroke/exhaustion and infection should also be explored. In most toxin induced hyperthermic syndromes, treatment includes benzodiazepine administration, active cooling and general supportive care. Antidotes may be attempted if the specific diagnosis is evident.

#### Electrocardiogram

Electrocardiographic (ECG) changes in the poisoned patient are commonly encountered [10]. Despite the fact that medications have widely varying indications for therapeutic use, many unrelated drugs share common cardiac electrocardiographic effects if taken in overdose. Toxins can be placed into broad classes based on their electrocardiographic effects (Table 2). The recognition of specific ECG changes associated with other clinical data (toxidromes) can lead clinicians to specific therapies that can be potentially life saving. Therefore, all seriously poisoned patients, particularly exposure to one of these agents is suspected, should have a minimum of an initial ECG. Repeat ECGs and cardiac monitoring would also be indicated if an ECG abnormality is identified or if the patient is at risk for delayed toxicity.

**Table 2:** Toxin Induced ECG Effects

**Toxins that prolong the QT interval**	**Toxins that prolong the QRS interval**
Antihistamines	Amantadine

Astemizole	Carbamazepine

Clarithromycin	Chloroquine

Diphenhydramine	Class IA antiarrhythmics

Loratidine	Disopyramide

Terfenadine	Quinidine

Antipsychotics	Procainamide

Chlorpromazine	Class IC antiarrhythmics

Droperidol	Encainide

Haloperidol	Flecainide

Mesoridazine	Propafenone

Pimozide	Citalopram

Quetiapine	Cocaine

Risperidone	Cyclic Antidepressants

Thioridazine	Amitriptyline

Ziprasidone	Amoxapine

Arsenic trioxide	Desipramine

Bepridil	Doxepin

Chloroquine	Imipramine

Cisapride	Nortriptyline

Citalopram	Maprotiline

Clarithromycin	Diltiazem

Class IA antiarrhythmics	Diphenhydramine

Disopyramide	Hydroxychloroquine

Quinidine	Loxapine

Procainamide	Orphenadrine

Class IC antiarrhythmics	Phenothiazines

Encainide	Medoridazine

Flecainide	Thioridazine

Moricizine	Propranolol

Propafenone	Propoxyphene

Class III antiarrhythmics	Quinine

Amiodarone	Verapamil

Dofetilide	

Ibutilide	

Sotalol	

Cyclic Antidepressants	

Erythomycin	

Fluoroquinolones	

Halofantrine	

Hydroxychloroquine	

Levomethadyl	

Methadone	

Pentamidine	

Quinine	

Tacrolimus	

Venlafaxine	

Studies suggest that approximately 3% of all non-cardiac prescriptions are associated with the potential for QT prolongation [11]. This drug induced QT prolongation may lead polymorphic ventricular tachycardia, most often as the torsades de pointes variant [12]. QT prolongation is considered to occur when the QTc interval is greater than 440 ms in men and 460 ms in women. The potential for an arrhythmia for a given QT interval will vary depending on the specific drug [13]. For example, venlafaxine is associated with QT prolongation, but rarely causes torsades due to venlafaxine-induced tachycardia. However, sotalol, on the other hand, induces bradycardia that increases the risk of torsades. Toxins may also inhibit fast cardiac sodium channels and thereby prolong the QRS complex [14]. The Na^+ ^channel blockers can cause slowed intraventricular conduction, unidirectional block, the development of a re-entrant circuit, and a resulting ventricular tachycardia or ventricular fibrillation. Myocardial Na^+ ^channel blocking drugs comprise a diverse group of pharmaceutical agents. There are multiple agents that can result in human cardiotoxicity and resultant ECG changes which may be treated through the administration of sodium bicarbonate. Physicians managing patients who have taken overdoses on medications should be aware of the various electrocardiographic changes that can potentially occur in the overdose setting.

#### Laboratory analysis

When evaluating the intoxicated patient, there is no substitute for a thorough history and physical exam. Samples cannot be simply processed by the lab with the correct diagnosis to a clinical mystery returning on a computer printout. Analytical capabilities vary significantly between regional care facilities and may limit the time in which results for analytical studies may be obtained which limits the use for direction of care in the acute setting [15]. When used appropriately, diagnostic tests may be of help in the management of the intoxicated patient. In the patient whose history is generally unreliable or in the unresponsive patient where no history is available, the clinician may gain further clues as to the etiology of a poisoning by responsible diagnostic testing. When a specific toxin or even class of toxins is suspected, requesting qualitative or quantitative levels may be appropriate if deemed necessary for diagnosis and treatment.

An acetaminophen (paracetamol) level drawn after a single, acute overdose is one of the few examples where a diagnostic laboratory result independent of clinical findings can be used to make treatment decisions [16-18]. Considering previous published studies, the authors recommended universal screening of all intentional overdose patients for the presence of acetaminophen. Because products containing salicylates are readily available, clinical effects of salicylate toxicity are non-specific, and a lack of metabolic acidosis does not rule out the potential for salicylate toxicity, clinicians should have a low threshold for also obtaining serum salicylate levels in potentially toxic patients [19].

The serum osmol gap is a common laboratory test that may be useful when evaluating poisoned patients. This test is most often discussed in the context of evaluating the patient suspected of toxic alcohol (*e.g. *ethylene glycol, methanol, and isopropanol) intoxication. Though this test may have utility in such situations, it has many pitfalls and limitations which limit its effectiveness. A calculated serum osmolarity (Osm_C_) may be obtained by any of a number of equations, involving the patient's glucose, sodium, and urea which contribute to almost all of the normally measured osmolality [20,21]. The most commonly utilized equation in the United States and Europe are noted below:



or



The difference between the measured (Osm_M_) and calculated (Osm_C_) is the osmol gap (OG): OG = Osm_M _- Osm_C_. If a significant osmol gap is discovered, the difference in the two values may represent the presence of foreign substances in the blood [22]. A list of possible causes of an elevated osmol gap is listed in Table 3. Traditionally, a normal gap has been defined as ≤ 10 mOsm/kg [23]. Unfortunately, what constitutes a normal osmol gap is widely debated [24-27]. There are several concerns in regard to utilizing the osmol gap as a screening tool in the evaluation of the potentially toxic-alcohol poisoned patient. If a patient's ingestion of a toxic alcohol occurred at a time distant from the actual blood sampling, the osmotically active parent compound may have been metabolized to acidic metabolites. The subsequent metabolites have no osmotic activity of their own and hence no osmol gap will be detected [20,28]. Therefore, it is possible that a patient may present at a point after ingestion with only a moderate rise in their osmol gap and anion gap [29,30]. However, recent research has found that an OG of 10 has a sensitivity of >85% and a specificity of <50% with a high negative predictive value (0.92) for identifying poisoned patients in which an antidote may be administered.(Lynd 08) Still, the osmol gap should be used with caution as an adjunct to clinical decision making and not as a primary determinant to rule out toxic alcohol ingestion. A "normal" osmol should be interpreted with caution; a negative study may, in fact, not rule out the presence of such an ingestion – the test result must be interpreted within the context of the clinical presentation. If such a poisoning is suspected, appropriate therapy should be initiated presumptively (*i.e. *ethanol infusion, 4-methyl-pyrazole, hemodialysis, *etc.*) while confirmation from serum levels of the suspected toxin are pending.

**Table 3:** Toxic causes of an elevated osmol gap

**Toxic alcohols**	Ethanol
	Isopropanol

	Methanol

	Ethylene Glycol

**Drugs/Additives**	Isoniazid

	Mannitol

	Propylene glycol

	Glycerol

	Osmotic contrast dyes

**Other Chemicals**	Ethyl ether

	Acetone

	Trichloroethane

Obtaining a basic metabolic panel in all poisoned patients is generally recommended. When low serum bicarbonate is discovered on a metabolic panel, the clinician should determine if an elevated anion gap exists. The formula most commonly used for the anion gap calculation is: [Na^+^] - [Cl^- ^+ HCO_3_]. This equation allows one to determine if serum electroneutrality is being maintained. The primary cation (sodium) and anions (chloride and bicarbonate) are represented in the equation [31]. There are other contributors to this equation that are "unmeasured" [32]. The normal range for this anion gap is accepted to be 8–16 mEq/L. Practically speaking, an increase in the anion gap beyond an accepted normal range, accompanied by a metabolic acidosis, represents an increase in unmeasured endogenous (*e.g. *lactate) or exogenous(*e.g. *salicylates) anions [33]. A list of the more common causes of this phenomenon are organized in the classic MUDILES pneumonic (Table 4). It is imperative that clinicians who admit poisoned patients initially presenting with an increased anion gap metabolic acidosis investigate the etiology of that acidosis. Many symptomatic poisoned patients may have an initial mild metabolic acidosis upon presentation due to the processes resulting in the elevation of serum lactate. However, with adequate supportive care including hydration and oxygenation, the anion gap acidosis should improve. If, despite adequate supportive care, an anion gap metabolic acidosis worsens in a poisoned patient, the clinician should consider either toxins that form acidic metabolites (i.e. ethylene glycol, methanol, or ibuprofen) or toxins which cause lactic acidosis by interfering with aerobic energy production (i.e. cyanide or iron) [34-36].

**Table 4:** Potential causes of increased anion gap metabolic acidosis

**M**ethanol
**U**remia

**D**iabetic ketoacidosis

**I**ron, **I**nhalants (i.e. carbon monoxide, cyanide, toluene), **I**soniazid, **I**buprofen

**L**actic acidosis

**E**thylene glycol, **E**thanol ketoacidosis

**S**alicylates, **S**tarvation ketoacidosis, **S**ympathomimetics

Many clinicians regularly obtain urine drug screening (UDS) on altered patients or on those suspected of ingestion. Such routine urine drug testing, however, is of questionable benefit for overdose and trauma in the emergency setting [37-40]. Most of the therapy is supportive and directed at the clinical scenario (i.e. mental status, cardiovascular function, respiratory condition), therefore the impact of such routine UDS is low. Interpretation of the results can be difficult even when the objective for ordering a comprehensive urine screen is adequately defined. Agents with very short half-lives such as gamma hydroxybutyrate (GHB) may be undetectable by laboratory analysis even in the acute setting. In contrast, when testing for agents with long half-lives, detection is possible but acuity may be difficult to predict. Most assays rely on antibody detection of drug metabolites with some drugs remaining positive days after use and thus may not be related to the patient's current clinical picture. The positive identification of drug metabolites is likewise influenced by chronicity of ingestion, fat solubility, and co-ingestions [41,42]. Conversely, many drugs of abuse are not detected on most urine drug screens, including GHB, fentanyl, and ketamine. The utility of ordering urine drug screens is fraught with significant testing limitations, including false-positive and false-negative results. Urine drug immunoscreening assays utilize monocolonal antibodies to detect structural conformations found in drugs belonging to a specific drug classes. Unfortunately, these antibodies have variable sensitivity and specificity [43]. Physicians need to be fully aware of the scope of drugs being detected and the sensitivity and specificity for the tests they are ordering. Many authors have shown that the test results rarely affect management decisions [15].

### Treatment approach

#### Decontamination

Decontamination of the severely poisoned patient must only be performed after careful consideration of the potential risks and benefits of the decontamination procedure. Although decontamination with ipecac, activated charcoal, gastric lavage and whole bowel irrigation were once common practice, current recommendations of the American Academy of Clinical Toxicology and the European Association of Poison Centers and Clinical Toxicologists reflect a trend towards more judicious use.

Syrup of ipecac is an agent that induces emesis through direct irritant action on the stomach and central action at the chemoreceptor trigger zone. Current recommendations discourage routine use of ipecac due to lack of evidence for improved outcomes and risks including delayed administration of oral antidotes and other decontamination products, aspiration, and complications from prolonged emesis and retching [44,45].

Activated charcoal is an agent possessing a large surface area that when administered orally, adsorbs ingested xenobiotics within the gastrointestinal track thereby preventing systemic absorption. Although it will adsorb most xenobiotics; some agents such as metals, ions and alcohols do not bind to charcoal. Charcoal is contraindicated in caustic ingestions because its presence in the gastrointestinal tract severely limits early endoscopic evaluation of caustic injuries. Charcoal aspiration events have been reported and careful attention should be given to the patient's ability to protect the airway prior to administration. If charcoal is to be administered by nasogastric tube, tube location should be confirmed by chest radiography prior to administration. Additional complications such as bowel perforation or obstruction following multidose charcoal administration have also been reported [46,47]. Overall, administration of activated charcoal remains a useful decontamination technique for patients presenting with early, potentially severe poisoning of absorbable xenobiotics provided risks are minimized [48].

Gastric lavage is the process of irrigating the gastric cavity to remove recently ingested material. Although liquid agents may be lavaged with a smaller diameter nasogastric tube, extraction of pill fragments requires use of a large bore tube (36–40 French). Large bore tubing may only be placed via the orogastric route to avoid trauma to the nasopharynx. Placement of an orogastric tube is a distressing procedure to perform in an awake patient and may be complicated by gagging and aspiration. Other serious complications such as hypoxia, laryngospasm, dysrhythmia and perforation have been also been reported. The procedure is contraindicated in cases of acid, alkali or hydrocarbon ingestion. Gastric lavage is not recommended for routine use in the poisoned patient [49].

Whole bowel irrigation pertains to the administration of a laxative agent such as polyethylene glycol to fully flush the bowel of stool and unabsorbed xenobiotics. Whole bowel irrigation is contraindicated in ileus, bowel obstruction or perforation, and in patients with hemodynamic instability. Although data is limited, whole bowel irrigation should be considered for substantial ingestions of iron, sustained release products, enteric coated products and symptomatic acute lead toxicity with known lead particles in the GI tract. In summary, although GI decontamination with activated charcoal and whole bowel irrigation may be of benefit particularly in early acute poisonings, it should only be attempted with careful consideration of the risks.

#### Seizures

Many toxins and withdrawal syndromes may result in seizures. The approach to toxin-induced seizure includes identification and management of hypoglycemia if present, maintenance of a patent airway, adequate oxygenation, prevention of injury, and administration of appropriate pharmacotherapy. For the toxin-induced seizure, benzodiazepine agents are the first line treatment of choice. Should benzodiazepines be ineffective, a second line agent such as a barbiturate may be employed. Propofol may also reduce seizure activity in intubated patients [50]. Phenytoin is generally not recommended in the severely poisoned patient as it is often ineffective and may worsen the overall toxicity of some agents[51]. In rare cases, pyridoxine (vitamin B_6_) is required for seizures induced by specific toxins, such as isoniazid or gyromitrin mushroom poisoning[52]. Investigation of other potential causes of seizure disorder such as intracranial hemorrhage or infarct through brain imaging should also be considered.

#### Antidotes

Although most poisonings are managed primarily with appropriate supportive care, there are several specific antidote agents that may be employed. Table 5 lists some of the more common antidotes for specific poisonings. A few antidotes are commonly utilized in the management of acute poisoning and deserve further discussion.

**Table 5:** Antidotes

**Agent or Clinical Finding**	**Potential Antidote(s)**
Acetaminophen	N-acetylcysteine

Benzodiazapines	Flumazenil

Beta blockers	Glucagon

Cardiac glycosides	Digoxin immune Fab

Crotalid envenomation	Crotalidae polyvalent immune Fab

Cyanide	Hydroxocobalamin

Ethylene glycol	Fomepizole

Iron	Deferoxamine

Isoniazid	Pyridoxine

Lead	Succimer Dimercaprol Calcium ethylenediamine tetra-acetic acid

Methanol	Fomepizole

Methemoglobinemia	Methylene blue

Monomethylhydrazine Mushrooms	Pyridoxime

Opioids	Naloxone

Organophosphates	Atropine Pralidoxime

Sulfonylureas	Glucose Octreotide

N-acetylcysteine (NAC) is an antidote that is used commonly in both early and late presentations of acetaminophen poisoning. It improves outcomes of acetaminophen poisonings by reducing the impact of the toxic metabolite of acetaminophen, NAPQI primarily through repletion of glutathione stores, enhancing NAPQI elimination, and reducing oxidative stress. Studies have shown that patients presenting with more severe hepatic injury due to late acetaminophen poisoning, may still benefit from NAC. Also, because NAC possesses few significant side effects it is frequently employed in the treatment of acetaminophen induced hepatic injury [53,54]. NAC can be given by both oral and intravenous administration. Oral dosing is 140 mg/kg loading dose followed by 70 mg/kg every 4 hours for 17 doses. Intravenous dosing consists of 150 mg/kg loading dose followed by 50 mg/kg over 4 hours followed by 100 mg/kg infused over 16 hours.

Opiate poisoning may be reversed with the opiate receptor antagonist naloxone. The preferred route of administration is via the intravenous route in order to facilitate careful dose titration [55]. Naloxone should be dosed to the desired endpoints until restoration of respiratory function, airway protection, and improved level of consciousness are achieved. Naloxone can precipitate profound withdrawal symptoms including agitation, vomiting, diarrhea, pilorection, diaphoresis, and yawning in patients chronically exposed to opiate agents. Administering naloxone through gradual 0.1 mg increments may reduce the risk of precipitating withdrawal symptoms. Naloxone's clinical effect may last for as little as 45 minutes. Therefore, patients exposed to methadone or sustained release opiate products are at risk for recurrence of narcotic effect. All patients requiring naloxone should be closely monitored for resedation for at least four hours after reversal with naloxone. If resedation occurs, it is reasonable to administer naloxone as an infusion. An infusion rate of 2/3 the effective initial bolus per hour is usually effective [55].

The benzodiazepine receptor antagonist flumazenil has also been employed to reverse the effects of severe benzodiazepine poisonings. While benzodiazepine overdose is rarely fatal when the sole ingestant, cases are often complicated with other central nervous system depressants (e.g., ethanol, opiates, and other sedatives) that may have synergistic activity. Flumazenil utility is limited by the risk of inducing benzodiazepine withdrawal in patients chronically exposed to benzodiazepines. Benzodiazepine withdrawal is of particular concern due to the potential for intractable seizures. Therefore, flumazenil should not be administered as a nonspecific coma-reversal drug and should be used with extreme caution after intentional benzodiazepine overdose [56]. Flumazenil finds its greatest utility for the reversal of benzodiazepine-induced sedation from minor surgical procedures or for exposures in other benzodiazepine naive patients, such as an accidental pediatric ingestion. The initial adult dose of flumazenil is 0.2 mg and should be administered intravenously over 30 sec. If no response occurs after an additional 30 sec, a second dose is recommended. Additional incremental doses of 0.5 mg may be administered at 1 min intervals until the desired response is noted or until a total of 3 mg has been administered. It is important to note that resedation may occur and patients should be observed carefully after requiring reversal.

Fomepizole (4-methylpyrazole) is a competitive alcohol dehydrogenase inhibitor administered in cases of suspected or confirmed ingestion of ethylene glycol or methanol. Fomepizole prevents the conversion of these agents to the metabolites associated with the majority of the toxic effects. Ethanol has also been used effectively as a competitive alcohol dehydrogenase inhibitor, however despite a significant cost increase, fomepizole use has become more frequent due to improved dosing, ease of administration and possible reduction in overall adverse events [57]. Fomepizole should be administered intravenously as a loading dose of 15 mg/kg, followed by doses of 10 mg/kg every 12 hours for 4 doses (48 hours) then 15 mg/kg every 12 hours thereafter; all doses should be administered as a slow intravenous infusion over 30 minutes [58]. During hemodialysis, the frequency of dosing should be increased to every 4 hours to account for removal of fomepizole during dialysis. Therapy should be continued until ethylene glycol or methanol concentrations are less than 20 mg/dL and the patient is asymptomatic [59].

### Enhancement of clearance/dialysis

In the severely poisoned patient, enhancing the toxin elimination may improve outcomes for some poisonings. Urine alkalinization may be considered for agents that are excreted as weak acids in the urine. By alkalinizing the urine through use of intravenous sodium bicarbonate, these weak acids will remain in a more polar ionized form in the urine that limits reabsorption and enhances elimination. Urine alkalinization may be considered for chlorpropamide, 2,4-dichlorophenoxyacetic acid, diflunisal, fluoride, methylchlorophenoxypropionic acid, methotrexate, phenobarbital and salicylates [60].

Dialysis may also be considered for poisons that are amenable to filtration across dialysis membranes [61]. These agents include agents that posses a low molecular weight, low volume of distribution, and low protein binding. Examples of agents that are commonly encountered and may require dialysis include salicylates, lithium, methylxanthines, and the toxic alcohols. Criteria for dialysis are variable across different types of poisonings. However, when considering hemodialysis, overall patient considerations such as the severity of symptoms and metabolic derangements should take priority in the decision making process over a specific drug level criteria. Drug levels may only estimate the level of pharmacodynamic response to toxins, and may guide decision-making but should not be used exclusively to determine dialysis needs.

## Conclusion

Ultimately, the management of the critically poisoned patient centers on careful supportive care. Care of the critically poisoned patient may be further maximized with appropriate decontamination, antidote administration, elimination enhancement and pharmaceutical interventions.

## Competing interests

The authors declare that they have no competing interests.

## Authors' contributions

JSB made substantial contributions to conception and design, acquisition of references, and manuscript revision. LKB made substantial contributions to conception and design, specifically focusing on the laboratory sections. CPH drafted the manuscript and revised it critically for important intellectual content. All authors read and approved the final manuscript.
